# Molecular Mechanisms of Hypericin and Hyperforin in Modulating Mammalian Neurotransmitter Systems: A Review

**DOI:** 10.1192/j.eurpsy.2025.1682

**Published:** 2025-08-26

**Authors:** D. J. Cox, R. Maldonado-Puebla, B. Carr

**Affiliations:** 1 Psychiatry, University of Florida, Gainesville, United States

## Abstract

**Introduction:**

Hypericin and hyperforin, key secondary metabolites of *Hypericum spp.,* commonly known as St. John’s Wort, are known for their ability to modulate neurotransmitter systems in the mammalian brain. These compounds, which evolved as plant defense chemicals, have significant implications for their interaction with mammalian neurobiology, particularly concerning serotonin, dopamine, and norepinephrine pathways.

**Objectives:**

This review aims to elucidate the precise molecular mechanisms by which hypericin and hyperforin influence mammalian brain function. The focus is on understanding how these compounds interact with neurotransmitter transporters and receptors, and how these interactions may lead to both therapeutic and adverse neurobiological outcomes.

**Methods:**

A comprehensive review of neurobiological and pharmacological literature was conducted, focusing on studies that detail the molecular interactions of hypericin and hyperforin with key components of neurotransmitter systems in mammals. in vitro binding assays, in vivo neuropharmacological studies, and molecular dynamics simulations were reviewed to understand these compounds’ binding affinities, receptor modulation, and downstream signaling effects.

**Results:**

Hypericin, with its planar polycyclic structure, exhibits a strong affinity for serotonin transporters (SERT), where it acts as a non-competitive inhibitor, leading to increased synaptic levels of serotonin. This mechanism mirrors that of selective serotonin reuptake inhibitors (SSRIs) but also introduces the potential for serotonin syndrome when combined with other serotonergic agents. Additionally, hypericin’s ability to generate reactive oxygen species (ROS) under light exposure contributes to neurotoxicity, particularly in regions of the brain exposed to higher oxidative stress.

Hyperforin, characterized by its phloroglucinol core and multiple prenyl groups, exerts its effects primarily through modulation of synaptic vesicle function and ion channel activity. It non-selectively inhibits the reuptake of several neurotransmitters, including serotonin, dopamine, and norepinephrine, through a mechanism involving transient receptor potential (TRP) channels. This broad-spectrum inhibition can lead to significant changes in synaptic plasticity and neurotransmission, impacting mood regulation, anxiety, and cognition.

**Conclusions:**

The interaction of hypericin and hyperforin with mammalian neurotransmitter systems underscores their potential as both therapeutic agents and neurotoxins. The molecular mechanisms by which these compounds modulate neurotransmitter transporters and receptors reveal a delicate balance between beneficial and adverse effects. Understanding these mechanisms is critical for evaluating the safety and efficacy of *Hypericum* extracts in clinical contexts, particularly regarding their impact on brain function and the potential for neurotoxicity.

**Disclosure of Interest:**

None Declared

